# Abrasive Wear, Scuffing and Rolling Contact Fatigue of DLC-Coated 18CrNiMo7-6 Steel Lubricated by a Pure and Contaminated Gear Oil

**DOI:** 10.3390/ma14227086

**Published:** 2021-11-22

**Authors:** Waldemar Tuszyński, Remigiusz Michalczewski, Edyta Osuch-Słomka, Andrzej Snarski-Adamski, Marek Kalbarczyk, Andrzej N. Wieczorek, Jakub Nędza

**Affiliations:** 1 Tribology Center, Łukasiewicz Research Network-Institute for Sustainable Technologies (L-ITEE), ul. Pułaskiego 6/10, 26-600 Radom, Poland; remigiusz.michalczewski@itee.lukasiewicz.gov.pl (R.M.); edyta.slomka@itee.lukasiewicz.gov.pl (E.O.-S.); andrzej.snarski@itee.lukasiewicz.gov.pl (A.S.-A.); marek.kalbarczyk@itee.lukasiewicz.gov.pl (M.K.); 2 Faculty of Mechanical Engineering, Kazimierz Pułaski University of Technology and Humanities, ul. Stasieckiego 54, 26-612 Radom, Poland; 3 Faculty of Mining, Safety Engineering and Industrial Automation, Silesian University of Technology, ul. Akademicka 2, 44-100 Gliwice, Poland; andrzej.n.wieczorek@polsl.pl; 4 Patentus SA, ul. Górnośląska 11, 43-200 Pszczyna, Poland; j.nedza@patentus.pl

**Keywords:** DLC coating, abrasive wear, scuffing, pitting, contaminated oil

## Abstract

Due to extreme working conditions of mining conveyors, which contaminate gear oil with solid particles, their transmissions are exposed to intensive abrasion, scuffing, and even rolling contact fatigue (pitting). These effects shorten gear life. To prevent their occurrence, a wear-resistant coating can be deposited on gear teeth. The resistance to abrasive wear, scuffing, and pitting was investigated and reported in the article. Simple, model specimens were used. Abrasive wear and scuffing were tested using a pin-and-vee-block tribosystem in sliding contact. A cone–three-ball rolling tribosystem was employed to test pitting. The material of the test specimens (pins, vee blocks, cones) was 18CrNiMo7-6 case-hardened steel. Two types of DLC (Diamond-like Coatings) coatings were tested, W-DLC and W-DLC/CrN. The vee blocks and cones were coated. Two industrial gear oils were selected to lubricate the specimens: one with a mineral and one with a synthetic PAO (polyalphaolephine) base, as pure oil or contaminated with solid particles from a coal mine. The results show that, to minimize the tendency to abrasion, scuffing, and pitting of specimens made of 18CrNiMo7-6 steel, the W-DLC/CrN coating should be deposited. This coating also gives very good protection when the lubricating oil is contaminated.

## 1. Introduction

Due to the very harsh conditions in coal and open-pit mines, gears of the transmissions of chain and belt conveyors are exposed to intensive abrasion, scuffing, and even rolling contact fatigue (pitting). This is caused by gears’ oil contamination with solid particles of coal or lignite. As a result, gear life is shortened. In extreme situations the weakened tooth may break, eliminating the transmission from service.

**Figure 1:**
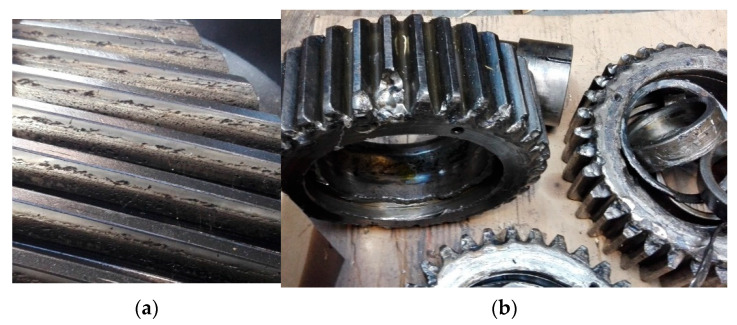
(**a**) Pitting on teeth flanks, (**b**) broken teeth.

Photographs of the gears of a transmission after service in a coal mine are shown in Figure 1.

To prevent the problem of intensive wear and to increase the life of gears in mining conveyors in this way, a thin, low-friction, wear-resistant coating can be deposited on the teeth [1]. Successful applications of coatings to prevent scuffing and micropitting are reported in the literature [2,3,4,5]. From the works of Beilicke et al. [6], as well as Liu et al. [7], it is apparent that, by the application of thin coatings, friction can be reduced. However, as it is shown in the works of Fujji et al. [8] and Michalczewski et al. [9,10], when the coating is used, pitting may be accelerated. Contradictory information is given in the works of Benedetti et al. [11], Singh et al. [12], and Szczerek et al. [13], because the authors report that the resistance to pitting may be improved when the coating is used.

Today, tested coatings are either non-DLC, or DLC coatings (DLC—diamond-like carbon). Examples of non-DLC coatings are include Nb-S [14], MoS_2_/Ti, C/Cr [15], TiN, and CrN [16]; whereas, DLC coatings are either doped: W-DLC [2], [16,17,18], Cr-DLC [19], Si-DLC [20]; or non-doped: a-C:H [19,20,21], ta-C [20]. The literature reports that DLC coatings are those that are most often tested for their tribological properties.

Much work has been published concerning the negative and abrasive action of solid contaminants, including the wear debris present in the oils. However, they concern mainly the lubrication of uncoated, steel specimens—see the works of Ludema [22], Enthoven and Spikes [23], as well as of Berg and Byheden [24]. Publications related to the prevention of wear by thin, wear-resistant coatings lubricated by contaminated oils are rarer. He et al. [25] tested the wear properties of DLC-coated bearing rollers. SiC particles were used as contaminants in the oil. The researchers have shown that wear-resistant coatings, when broken up by hard contaminants, can damage the rubbing surfaces. On the opposite side, Michalczewski et al. [26], on the base of scuffing tests using a pin-and-vee-block tribotester and the oil contaminated with dust composed of mostly SiO_2_ and Al_2_O_3_, have stated that the resistance to scuffing of DLC-coated samples improved significantly compared with the results for the uncoated tribosystem.

Concerning testing methods, screening tests are often performed first. They are carried out using simple, model specimens. Then the second phase of testing begins, i.e., component tests on gears, for example, are performed to verify the results obtained from the tests on model specimens. This is because component tests are very long and expensive; pitting tests carried out on gears may require even months to complete. This approach can be found in the works of Lacey [27], Van de Velde et al. [28,29] Bisht and Singhal [30], as well as Trzos, Szczerek, and Tuszyński [31]. As concerns the tribological investigation of thin coatings, most tests, especially concerning pitting, are performed using simple, model specimens [16,32,33,34,35,36].

In this paper, the effects of two types of commercially available DLC coatings is presented. The DLC coatings were chosen because they exhibit low coefficients of friction [37], which is important in, e.g., transmissions of belt conveyors.

Previously, the authors of this paper performed research on simple model specimens working in sliding or rolling contact. The following coatings were tested: TiN, CrN, W-DLC, MoS_2_/Ti [16], and W-DLC/CrN [36]. The tested oils were mineral, synthetic, and non-toxic (vegetable) oils. The aim of the experiments was to find an effects of different kinds of lubricating oil on various modes of wear.

The authors of this paper also performed tests on coated gears. In work [4], the results of testing the scuffing of bevel gears are presented. A W-DLC coating was deposited on the wheel. In works [10,11], the results of testing the pitting of W-DLC-coated and MoS_2_/Ti-coated spur gears are published. The aim was to examine the effects of four material combinations on their resistances to pitting, which were an uncoated wheel and pinion, a coated wheel and pinion, a coated wheel and an uncoated pinion, as well as an uncoated wheel and a coated pinion.

A novelty of this paper is its direct comparison of W-DLC and W-DLC/CrN coatings, as well as having also performed tests with contaminated lubricating oil. The W-DLC coating in the previous authors’ works exhibited the most satisfactory results, while W-DLC/CrN, with a CrN layer in its microstructure, was presumed to give a better resistance to pitting than W-DLC. This is why these coatings were selected for the present research. They are compared for their resistances to abrasion, scuffing, and pitting. The experiments were performed using simple model specimens made of 18CrNiMo7-6 case-hardened steel.

The results obtained made it possible to select one of these two coatings for further experiments—with a contaminated oil. The results show that, to minimise the tendency to abrasion, scuffing, and pitting of specimens made of 18CrNiMo7-6 steel, a W-DLC/CrN coating should be deposited. This coating also gives very good protection when the lubricating oil is contaminated.

The research finally aims at extending the life of the gears in mining transmissions of chain and belt conveyors in the mining industry.

## 2. Materials and Methods

### 2.1. Test Specimens

**Figure 2:**
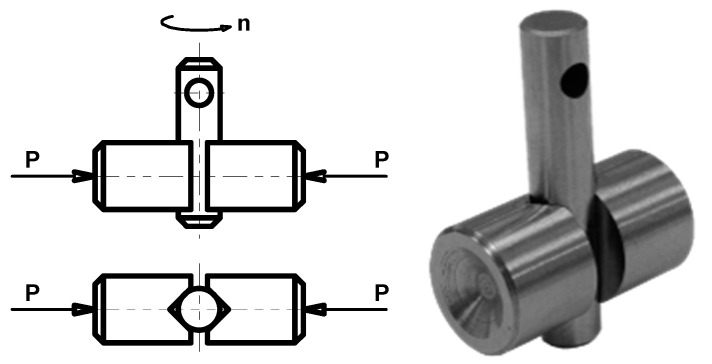
Pin and vee block tribosystem; P-load, n-speed.

The tribosystem used in the abrasion and scuffing tests is shown in Figure 2.

Two vee blocks are pressed at the load P to the test pin. The test pin rotates at the speed *n* = 290 rpm, which is constant. The shear pin is located in the hole of the pin, and it transmits the driving torque from test shaft. The lubricating oil is poured into the reservoir, and the whole contact area is immersed in the oil. The heater located in the reservoir makes it possible to increase the initial oil temperature up to 70 °C. Such a temperature is expected in the transmissions of mining conveyors.

In the abrasion and scuffing tests, the vee blocks were coated, leaving the test pins uncoated.

**Figure 3:**
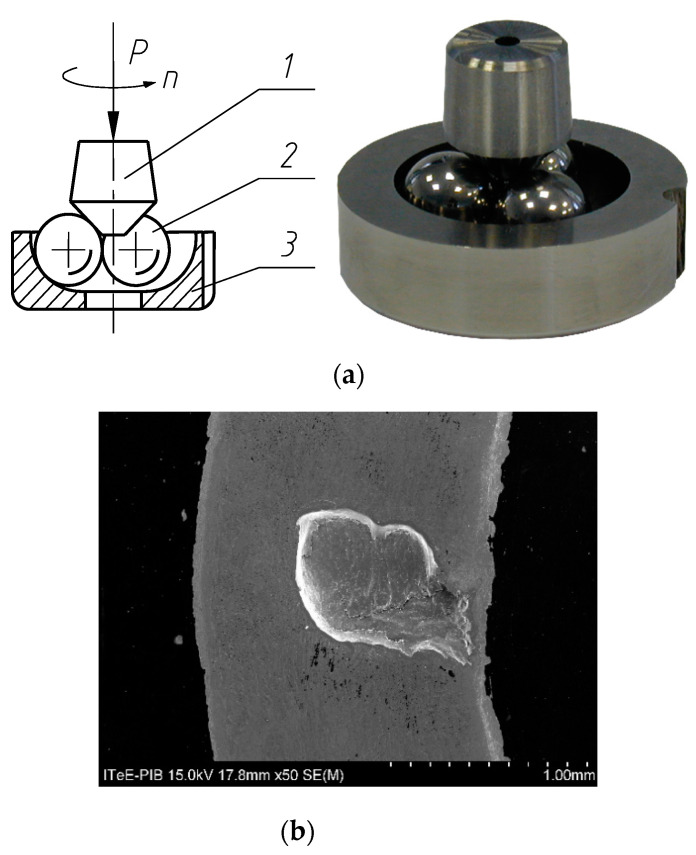
(**a**) Cone–balls tribosystem, (**b**) SEM image of a pitted cone; P-load, n-speed.

In the pitting tests a cone–three-ball tribosystem was employed—Figure 3. In the figure, the SEM image of a pitted cone is also shown.

Three balls (Figure 3a, left, 1) rotate in the race (Figure 3a, left, 2). They are pressed against the cone (Figure 3a, left, 1) at the load P. The cone rotates at speed n. The tribosystem is immersed in oil. The initial temperature of the oil was of 70 °C, as in the abrasion and scuffing tests.

In the pitting tests, the test cones were coated.

### 2.2. Substrate Materials

A 18CrNiMo7-6 case-hardened steel was used to make the test pins, vee blocks, and cones. This steel is intended for the manufacturing of gears for the transmissions of mining conveyors.

In the pitting tests, the material of the balls and races was 100Cr6 bearing steel.

The hardness of the test pins, vee blocks, and cones was 62 HRC (Rockwell hardness C). The roughness, Ra, of the vee blocks and cones was 0.20 μm. The roughness of the pins was 0.52 μm.

### 2.3. Coatings

Two types of antifriction DLC coatings were selected for testing. Both of them represent an a-C:H:Me group. Their structures were W-DLC and W-DLC/CrN (more details can be found in Section 3.1). They were deposited by reactive sputtering in the physical vapour deposition (PVD) process, by Oerlikon Balzers Coating Poland Sp. z o.o., Tczew, Poland.

### 2.4. Lubricating Oils

Two commercial industrial gear oils were selected for lubrication, namely, a mineral and a synthetic one, having a PAO (polyalphaolephine) base—Fuchs Renolin CLP 320 and Shell Omala S4 GX 320.

Both the oils are classified to the same viscosity grade—VG 320. However, their viscosity indexes (VI) significantly differ. For the mineral oil, VI = 95, and for the PAO oil VI = 159. Both the oils are used, e.g., to lubricate gears in transmissions of mining conveyors.

Mineral oil was used in the first phase of the experiment (tribological behaviour of the DLC coatings—Section 3.2).

In the second phase of the experiment, the synthetic oil was used (testing using contaminated oil—Section 3.3).

### 2.5. Test Methods

Procedure A of the ASTM D 2625, slightly modified, was followed in the abrasion tests. It was performed in the following steps:

− a run-in phase at 1334 N for 3 min;
− the first phase of the test at 2224 N—for 1 min or up to failure;
− if no failure is observed, the second phase is performed at 3336 N—for 1 min or up to failure; and
− if no failure is observed, the third phase is performed at 4448 N until failure occurs.

Failure appears when the following occur:

− a sharp rise of the friction torque by 1.13 Nm occurs above the steady-state value;
− the shear pin breaks;
− maintaining the load is impossible; or
− reaching a total time of 10,000 s excluding 3 min (run-in).

The result of the test is the endurance (wear) life, i.e., the total time before failure, excluding the run-in period. The run was repeated a minimum of three times. The endurance (wear) life is a measure of the resistance to abrasive wear.

In scuffing tests the ASTM D 3233, the Method A procedure was followed. It was performed in two steps:

− a run-in phase at 1334 N for 5 min; then
− the regular test, in which the load continuously increases until failure occurs or until the maximum load is reached.

This approach creates very harsh conditions in the friction zone, differentiating the scuffing test from the abrasion one, where the load is changed in steps.

Failure appears when:

− the shear pin breaks; or
− the test pin breaks.

The result of the test is the load at failure or a maximum attainable load, which are measures of the resistance to scuffing. The run was repeated a minimum of four times.

To test pitting, IP 300 standard was followed. The tests were performed under the following conditions:

− a rotational speed of 1450 rpm;
− an applied load of 3924 N (400 kgf); and
− the run duration, until pitting occurs.

The required number of valid runs is 24. The condition necessary to classify the run as valid is that pitting occurs on the cone. The time to pitting occurrence was measured in each run.

The 24 measured pitting failure times are then presented in Weibull co-ordinates; they are the estimated cumulative percentage failed versus the time to pitting failure. The fatigue life L_10_, being a measure of the resistance to pitting, is determined from the line fitted to the points in Weibull co-ordinates. The L_10_ is the life at which one would expect that ten per cent of a large number of test cones to fail.

### 2.6. Tribological and Analytical Instruments

For tribological testing the following devices were used:

− a T-09 pin and vee block tribotester produced by the Łukasiewicz—Institute for Sustainable Technologies, Radom, Poland; it was used to test the resistance to abrasive wear and scuffing;
− a T-02U four-ball testing machine produced by the Łukasiewicz—Institute for Sustainable Technologies, Radom, Poland; it was used to determine the resistance to pitting and according to the test method described in the work [36], the top ball was replaced with a cone.

For the coating characterisation the following instruments were used:

− a JY 10000 RF glow discharge optical emission spectrometer (GDOES) produced by Jobin Yvon Horiba, Palaiseau, France, for depth profiling (qualitative analysis at the pressure of 600 Pa and power of 30 W);
− a CALOWEAR calotester, produced by CSM, Peseux, Switzerland, for the measurement of the coating thickness (100Cr6 steel ball of 25.4-mm diameter, shaft speed of 400 rpm, 500-m distance);
− a REVETEST scratch tester, produced by CSM, Peseux, Switzerland, for the measurement of adhesion (Rockwell C-type indenter, linearly increasing load from 0 to 100 N, constant load increasing rate of 10 N/mm);
− a NanoHardness Tester, produced by CSM, Peseux, Switzerland (Berkovich indenter, indenter cavity not exceeding 10% of the coating thickness, load of 5 mN); and,
− a Form Talysurf PGI 830 stylus profilometer, produced by Taylor Hobson, Leicester, UK, for the measurement of the coating roughness (stylus radius of 2 μm, Gaussian filtering).

The surface was also analysed using the following:

− a SU-70 field-emission scanning electron microscope (FE-SEM), produced by Hitachi, Tokyo, Japan, integrated with an NSS 312 energy dispersive spectrometer (EDS) produced by Thermo Scientific, Madison, Wisconsin, USA, for surface imaging in microscale and elemental analysis (acceleration voltage of 15 kV, take-off angle of 30°);
− a D8 DISCOVER X-ray diffractometer (XRD) produced by Bruker, Karlsruhe, Germany, for determination of the crystal lattice deformation, based on which the residual (subsurface) stresses in the samples were calculated by means of the Leptos 7.6 Stress software with an implemented Sin^2^ψ method (cobalt X ray tube, generator power of 1400 W);
− a CCI optical profilometer, produced by Taylor Hobson, Leicester, UK, for 3D surface imaging at the micro scale and the measurement of surface roughness (10× magnification);
− a Q-scope 250 atomic force microscope (AFM) produced by the Quesant Instrument Corporation, Agoura Hills, USA, or 3D surface imaging at the nano scale (contact-mode measurement); and,
− a MM-40 optical microscope produced by Nikon, Tokyo, Japan (100× magnification);
− an FM-800 Series microhardness tester produced by FUTURE TECH Corp., Tokyo, Japan (Vickers indenter, load of 100 gf).

Before analyses, the test specimens were washed ultrasonically with n-hexane, and then dried in the open air.

### 2.7. Statistical Analysis

A statistical analysis was also carried out. After the abrasion and scuffing tests, confidence intervals at 95% probability were determined. After the pitting tests, the scatter of results was represented by confidence intervals calculated at 90% probability.

## 3. Results and Discussion

### 3.1. Coating Characterisation

To facilitate the coating characterisation, ‘control’ disks were manufactured. The disks had the flat surface roughness identical to the rubbing surfaces of the test specimens (vee blocks and cones), i.e., Ra = 0.2 μm. They were made of identical material, i.e., 18CrNiMo7-6 case-hardened steel. The disks were coated together with the test specimens and the coating was deposited on their flat surface.

**Figure 4:**
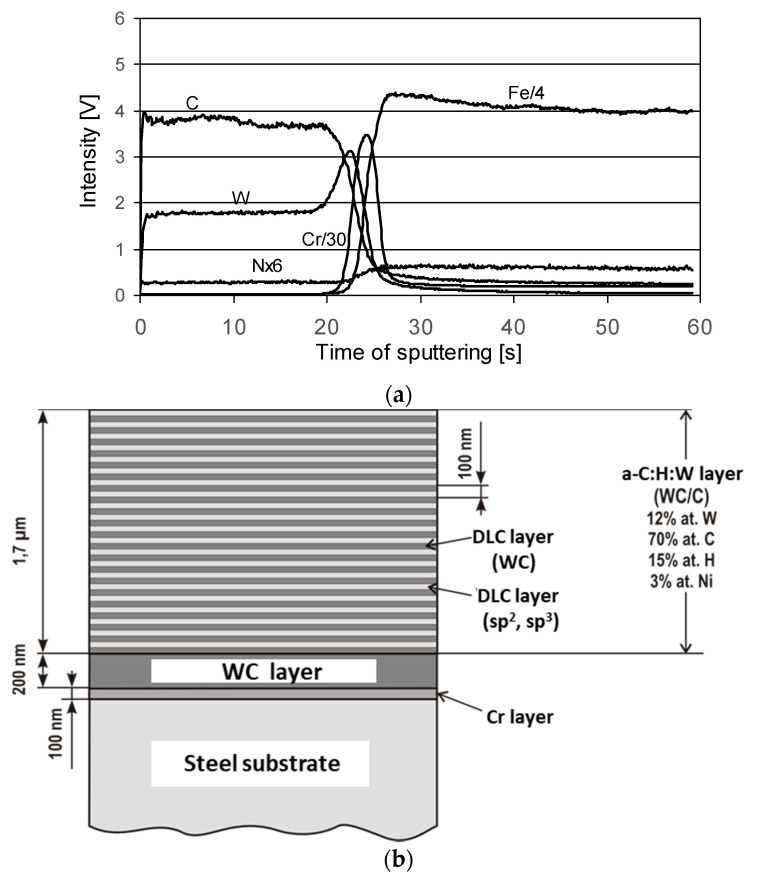
(**a**) GDOES depth profile of W-DLC coating, (**b**) model of W-DLC microstructure [38].

**Figure 5:**
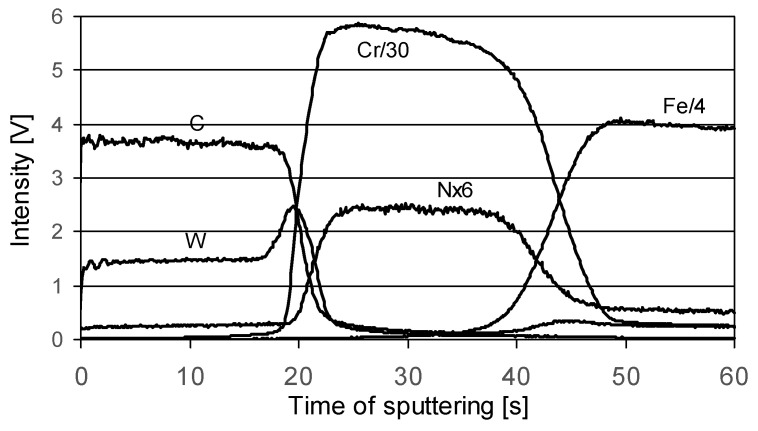
GDOES depth profile of W-DLC/CrN coating.

The microstructure of the coatings revealed in glow discharge optical emission spectrometry (GDOES) analyses are illustrated in Figure 4 and Figure 5. In the figures, intensity is expressed in volts, which indirectly relates to the concentration of each element in a qualitative analysis.

The W-DLC coating consists of three layers [39]. A layer of chromium with a thickness of 100 nm is deposited on the steel substrate. It is an interlayer that improves the adhesion of the next layer of tungsten carbide (WC) to the steel substrate. Hard WC (approx. 200 nm thick), in the case of wearing off the outer layer, protects the substrate against abrasion, hence, the very good antiwear properties of the coating.

The outer layer is responsible for the interaction with the rubbing element. It is a multilayer of a cyclically repeated (approx. every 100 nm) structure (commonly known as WC/C), which includes a WC-rich DLC layer and a DLC layer. WC is in the form of nanocrystals with diameters of approximately 2 nm, randomly distributed in the DLC layer. Its good antifriction properties result from the high content of amorphous carbon (sp^2^) in the outer layer of the coating. As was shown in work [37], under conditions of dry friction against steel, a low coefficient of friction, typical for a lubricated contact, is obtained, i.e., below 0.1.

The W-DLC/CrN coating is the modification of the former one. Figure 5 shows that the coating consists of three main layers: very ductile CrN on the substrate, WC, and then WC/C. The CrN layer is presumed to give very good support properties, especially in cyclic loaded contacts.

The basic properties of the W-DLC and W-DLC/CrN coatings are the following, respectively: thicknesses of 1 and 2 μm, adhesions in scratch tests of 90 and 101 N, nanohardnesses of 15.5 and 16 GPa, and roughnesses Ra of 0.12 and 0.12 μm.

It should be mentioned, here, that in the literature on the subject, researchers have reported attempts to create a DLC coating with specific properties, e.g., in the work [39], wherein special attention was paid to obtaining the sp^3^ structure.

### 3.2. Tribological Behaviour of the DLC Coatings

In this phase of the experiment, mineral oil was used for lubrication.

#### 3.2.1. Abrasion Tests

**Figure 6:**
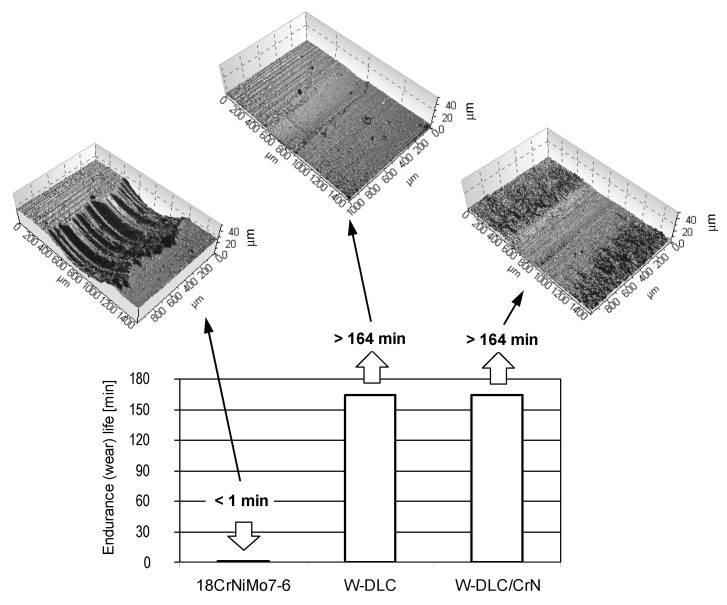
Endurance (wear) lives obtained in the abrasion tests.

The results obtained for the three material combinations in the abrasion tests, together with optical profilometric images of the wear scars on the vee blocks, are illustrated in Figure 6.

By depositing the thin, antifriction coating on one specimen, the resistance to abrasion notably rises. This is accompanied by much less wear of the coated blocks. Moreover, the worn surface of the coated vee block is much smoother than in case of the uncoated one. This shows an effect of the hard WC interlayer in both coatings, giving the very good resistance to abrasion.

#### 3.2.2. Scuffing Tests

**Figure 7:**
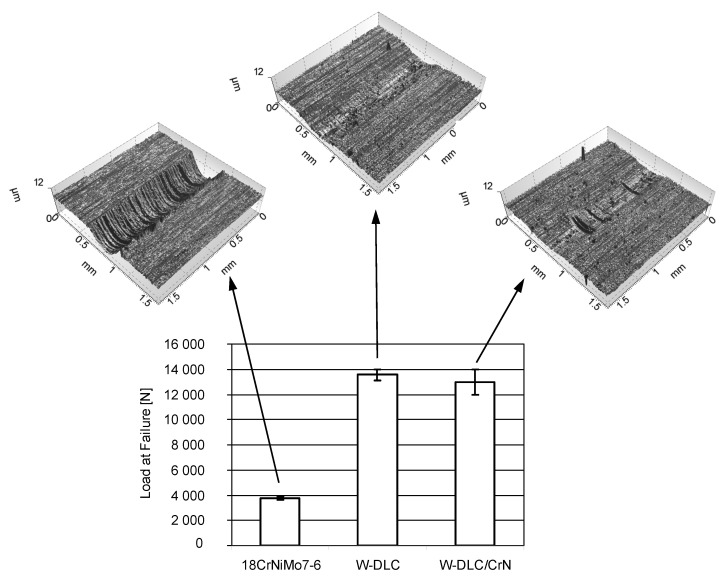
Loads at failure obtained in the scuffing tests.

The results obtained for the same three material combinations in the scuffing tests, together with optical profilometric images of the wear scars on the vee blocks, are shown in Figure 7.

By depositing the thin, antifriction coating on one specimen, the resistance to scuffing significantly rises. This is a result of a smaller tendency to create adhesive bonds (lower affinity) in case of two different rubbing materials than when the contact is uncoated. Another reason is related to the hardness. The high hardness of the WC layer in the coatings also prevents the creation of adhesive bonds. Consequently there is less friction, and thus less-intensive scuffing propagation, because the wear scars of the coated vee blocks are much smaller than in case of the uncoated one.

As in the abrasion tests, no significant difference between the coatings was found; the confidence intervals in Figure 7 overlap each other, and the wear is comparable.

#### 3.2.3. Pitting Tests

**Figure 8:**
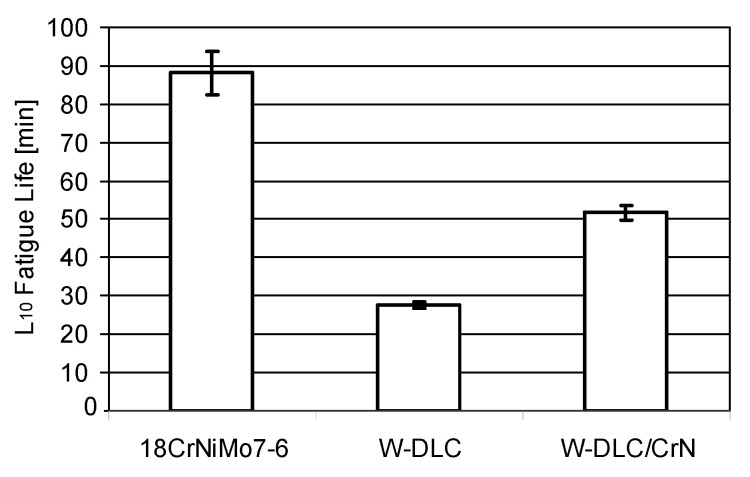
L_10_ Fatigue Lives obtained in the pitting tests.

The results obtained for the same three material combinations in the pitting tests, are illustrated in Figure 8.

When the coating is deposited, the resistance to pitting significantly drops. However, the W-DLC/CrN coating shows much higher fatigue life than W-DLC.

A general reason for a drop in the pitting resistance of the coated samples can be related to defects in the coating surface and the subsurface stress.

**Figure 9:**
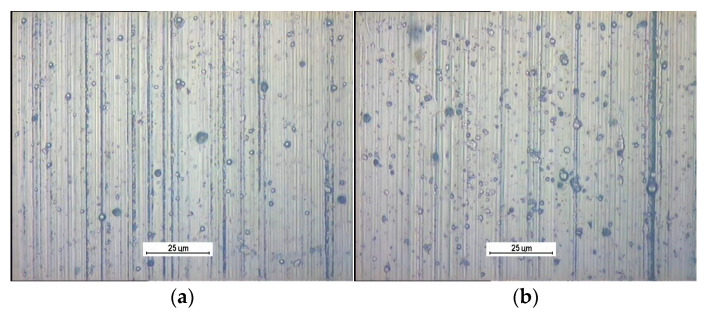
Optical microscope images of the surface of the coatings: W-DLC (**a**), W-DLC/CrN (**b**).

**Figure 10:**
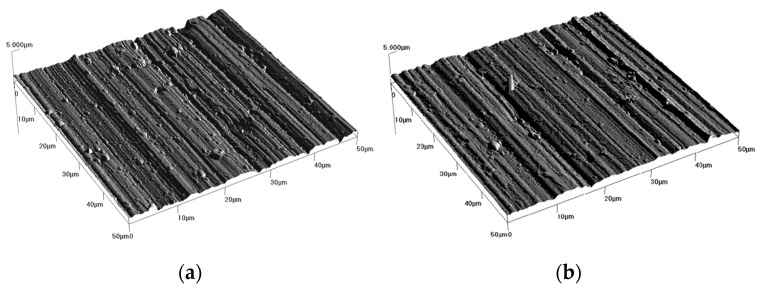
AFM images: of the surface of the coatings: W-DLC (**a**), W-DLC/CrN (**b**).

The coating surface observed using an optical microscope and an atomic force microscope is shown in Figure 9 and Figure 10. The parallel grooves come from the grinding process of the steel samples.

Numerous droplets on the surfaces of the coatings can be considered defects, which are responsible for pitting initiation.

**Figure 11:**
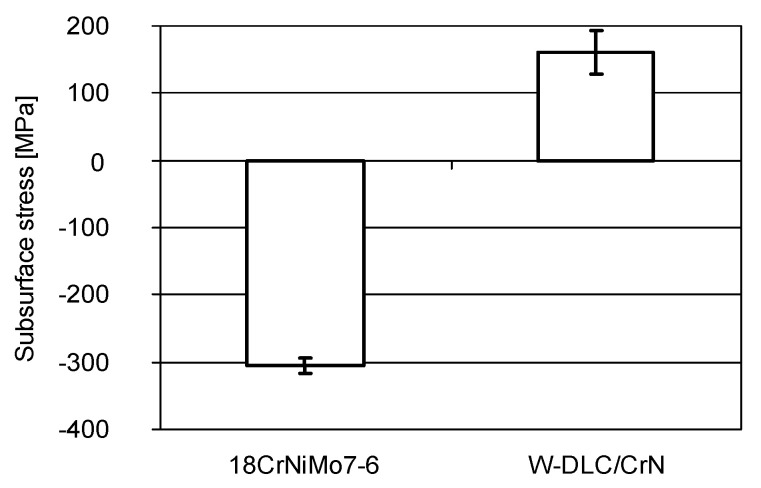
Subsurface stress after deposition of W-DLC/CrN coating, determined using XRD.

The subsurface stress in the surface layer was determined for the W-DLC/CrN coating—after its deposition on the substrate material—Figure 11. The measurement was performed at three different points. The confidence intervals calculated at 95% probability are included in the graph.

From Figure 11, it appears that, for the coated samples, the beneficial compressive stresses (with the minus sign) have changed into tensile stresses, which has a negative effect on fatigue life. This was also found in other works. Vackel and Sampath [40] state that coatings increase fatigue life when they possess high compressive residual stress, hardness, and microstructural density. On the opposite side, when coatings exhibit tensile residual stress, pitting may be accelerated. Varis et al. [41] also observed that compressive stress in the surface resulted in good fatigue behaviour.

**Figure 12:**
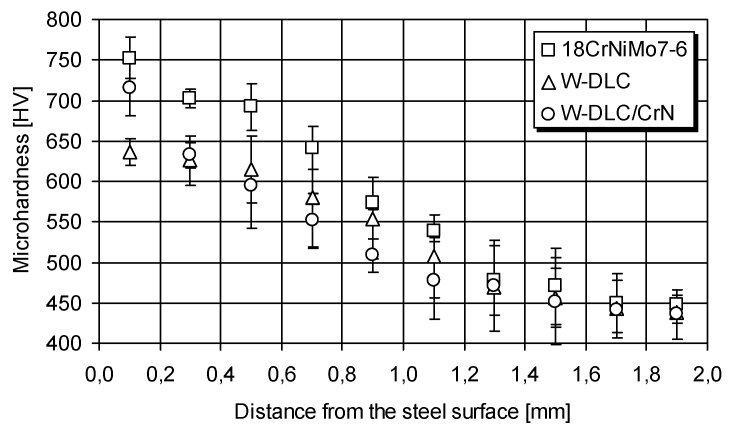
Microhardness profiles after deposition of W-DLC and W-DLC/CrN coating.

Finally, to reveal the reason why the W-DLC/CrN coating shows much higher fatigue life than W-DLC, the microhardness profiles were determined—Figure 12. The microhardness was measured along three different lines. The confidence intervals calculated at 95% probability are included in the graph.

As can be seen, during the process of coating deposition at a temperature of about 220–250 °C, the microhardness dropped below the level observed for the uncoated steel. This results from phase transformations occurring in the steel substrate. At the zone near the surface—down to 0.2 mm—which is most important in fatigue initiation, the microhardness under W-DLC/CrN was higher than under W-DLC. This remained correlated with the resistance to pitting shown in Figure 8; the higher the microhardness near the surface, the longer is the fatigue life. This observation is supported by other research, e.g., by Piekoszewski [42].

Another possible reason may be related to adhesion. For the W-DLC and W-DLC/CrN coatings, the adhesion determined in scratch tests was 90 and 101 N, respectively. Thus, W-DLC/CrN coating adheres stronger to the substrate than W-DLC; hence, it is better resistance to pitting than for the W-DLC coating.

In summary, in comparison with the uncoated specimens, both tested coatings improved the resistance to abrasion and scuffing in the same way. However, the W-DLC/CrN coating exhibited better resistance to pitting than did W-DLC, although both coatings reduced the resistance to pitting in comparison with the uncoated tribosystem. Thus, for the second phase of the experiments, a W-DLC/CrN coating was selected as best-suited for deposition on 18CrNiMo7-6 case-hardened steel.

It is necessary to comment on the methodology of pitting testing. The tests, according to IP 300, are to be performed under one load. As other experiments have shown [13], it is necessary to perform tests under different loads and then build and analyse the S–N curve to determine the stress level corresponding to infinite life. This stress level best represents fatigue life.

### 3.3. Testing Using Contaminated Oil

For the second phase of the experiment, PAO oil was selected for the lubrication of the W-DLC/CrN-coated 18CrNiMo7-6 steel specimens; PAO oils exhibit a much better viscosity index than mineral ones, which was a decisive reason for such a selection, given the harsh conditions of mining conveyors’ work.

The gear oil with a PAO base was contaminated at a concentration of 1.5% by weight with the dust obtained from a coal mine. Its main content was carbon (50%) and silica. The maximum particle size was 70 μm.

**Figure 13:**
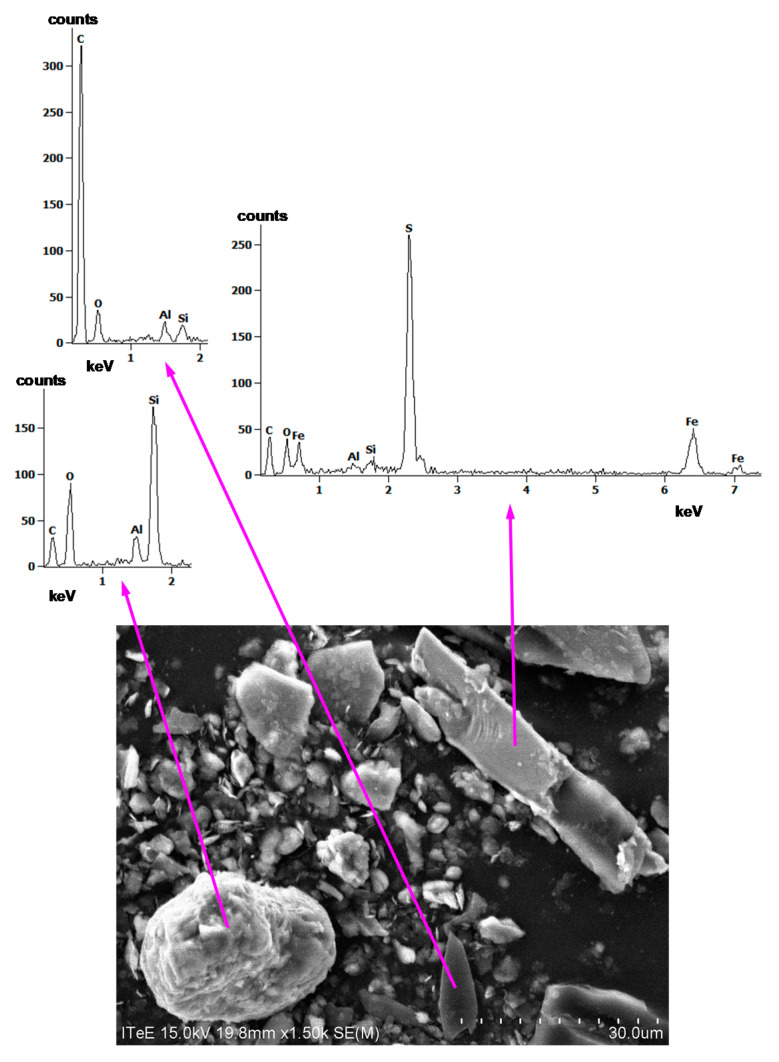
SEM image of carbon, silica, and iron sulphide particles, together with EDS spectra.

**Figure 14:**
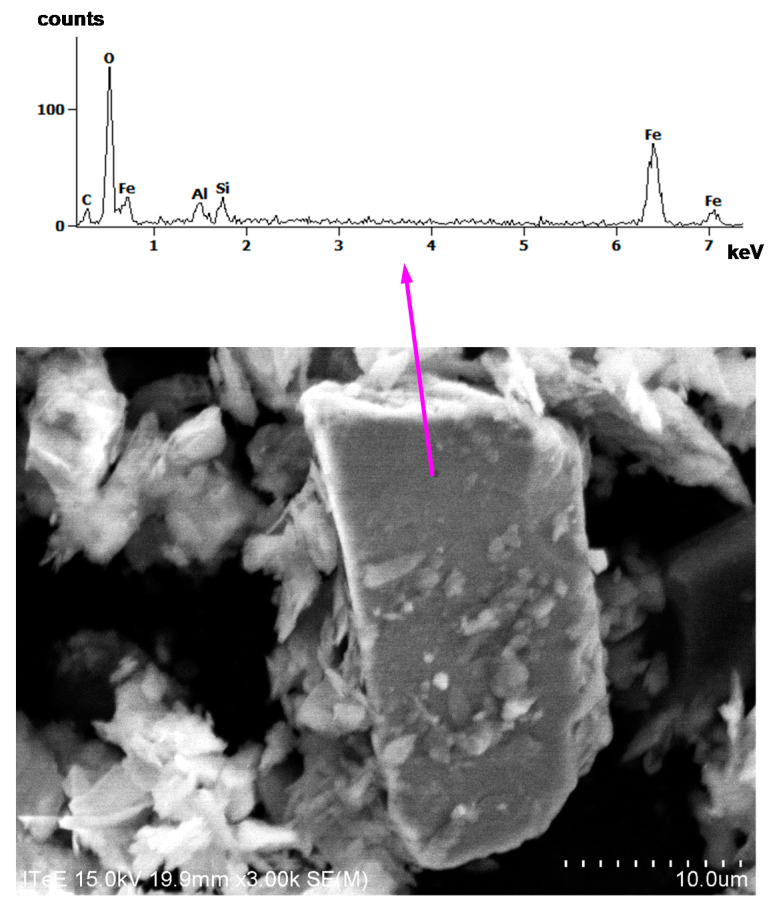
SEM image of iron oxide particles, together with EDS spectrum.

SEM images of some selected particles, together with EDS spectra, are shown in Figure 13 and Figure 14.

By analysing elements from the EDS spectra in Figure 13 and Figure 14, it is apparent that the particles are carbon, silica, alumina, iron sulphide, and iron oxide. Aluminium, in all spectra, may, however, have also originated from the stub in the SEM chamber on which the dust was spilled before the analyses.

#### 3.3.1. Abrasion Tests

**Figure 15:**
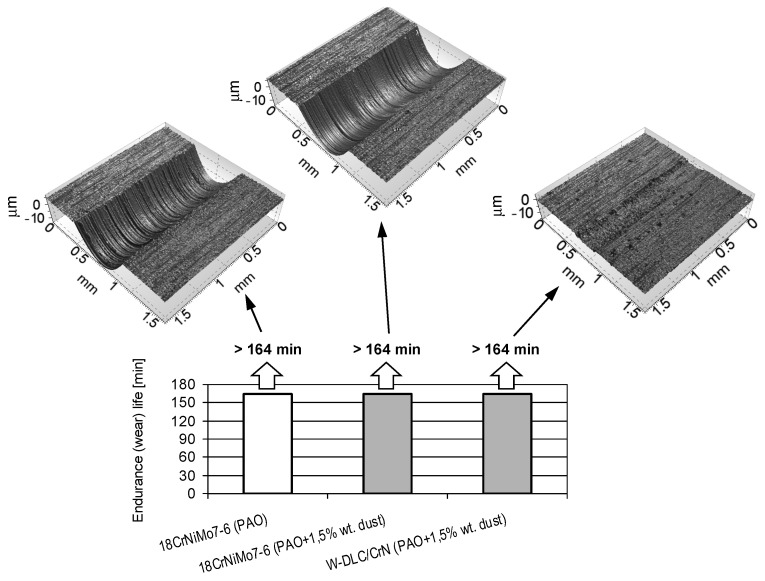
Endurance (wear) lives obtained in the abrasion tests.

The results of the abrasion tests are presented in Figure 15. The figure compiles the results from the uncoated tribosystem lubricated with the PAO oil, the uncoated tribosystem lubricated with the contaminated PAO oil, and the coating–steel tribosystem lubricated with the contaminated PAO oil. Optical profilometric images of the wear scars on the vee blocks are also shown in the figure.

From Figure 15, it appears that the coating–steel material combination allows the maximum value of the resistance to abrasive wear to be obtained, identically to the uncoated tribosystems, and the presence of dust is irrelevant. Thus, to reveal a possible difference, an additional criterion was checked. In the upper row of Figure 13 are 3D images of the wear scars.

As can be observed when the W-DLC/CrN coating is deposited on one specimen, the wear of the coated vee block lubricated with the contaminated oil is incomparably less than in case of the uncoated specimens, even when lubricated with the pure oil. This is an effect of the presence of the hard—and, hence, resistant to abrasion—WC layer in the coating.

#### 3.3.2. Scuffing Tests

**Figure 16:**
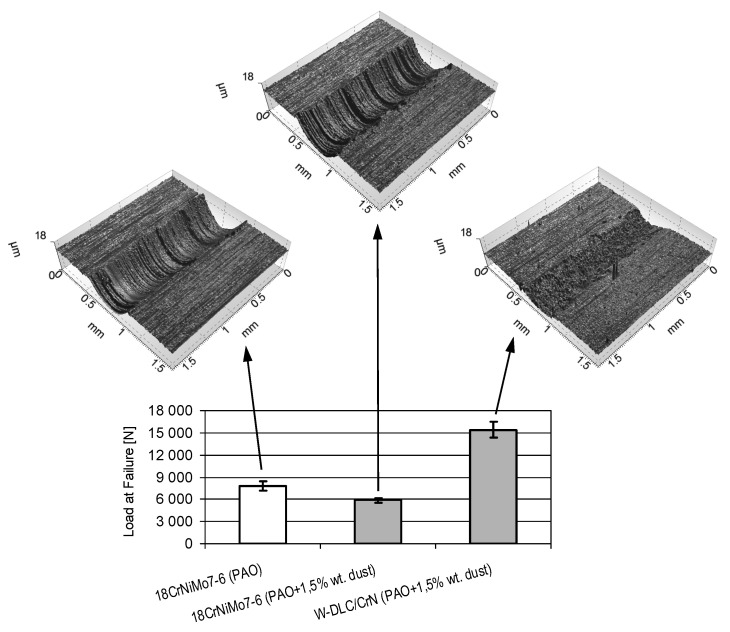
Loads at failure obtained in the scuffing tests.

The results of the scuffing tests are presented in Figure 16. Optical profilometric images of the wear scars on the vee blocks are also shown in the figure.

From Figure 16 it can be observed that, despite the contamination of the oil, a much higher resistance to scuffing is observed for the coating–steel tribosystem than for the oil without impurities, lubricating the uncoated tribosystem. This is accompanied by much less wear of the coated vee block in comparison with the uncoated one. The lowest resistance to scuffing is observed when contaminated oil lubricates the uncoated tribosystem. This means that the application of the coating allows the compensation of the potential drop in scuffing resistance caused by oil contamination, and even increases it.

The beneficial behaviour of the coating is a result of a smaller tendency to create adhesive bonds (lower affinity) in the case of two different rubbing materials than when the contact is uncoated. Another reason is related to hardness. The high hardness of the WC layer in the coatings also prevents the creation of adhesive bonds. As an effect, the wear scar of the coated vee block was small, in spite of its oil contamination, which was much smaller than in case of the uncoated one, even when the tribosystem is lubricated with pure oil.

#### 3.3.3. Pitting Tests

**Figure 17:**
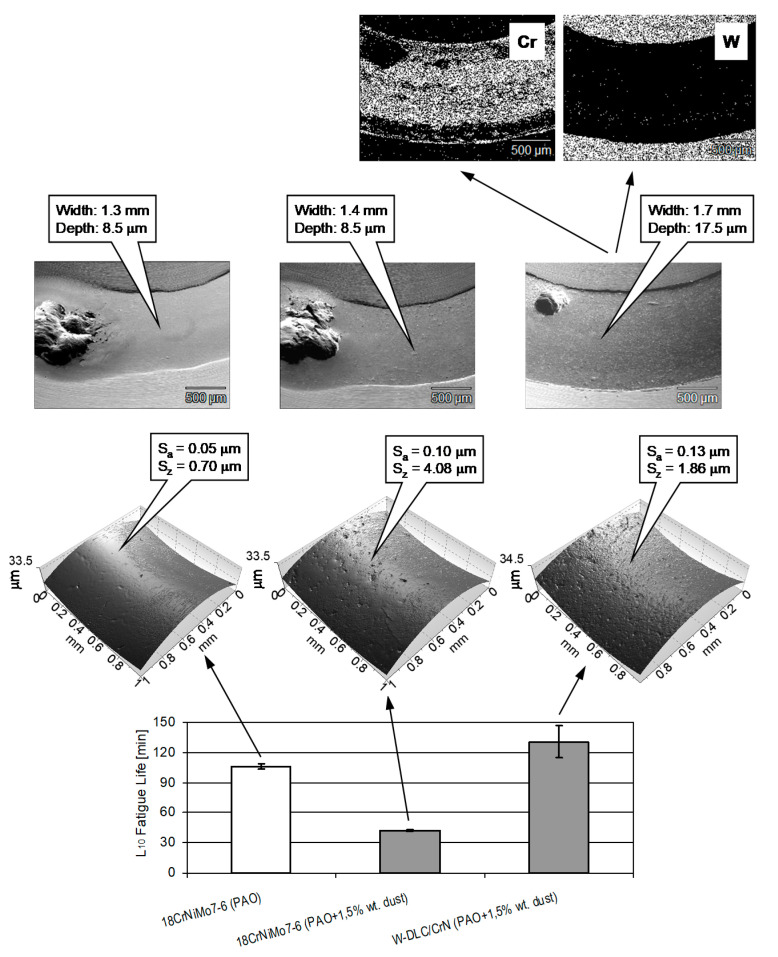
L_10_ Fatigue lives obtained in pitting tests as well as optical profilometric images of the wear tracks on the test cones (**bottom** row), SEM images of the wear track (**middle** row), and EDS maps of the wear track on the coated cone (**upper** row).

The results of the pitting tests are presented in Figure 17.

From Figure 17, it can be stated that, despite oil contamination, a higher pitting resistance is observed for the steel–coating tribosystem than for the uncoated contact lubricated with the oil without impurities. The worst result was observed from the contaminated oil lubricates in the uncoated tribosystem. This means that the application of the coating compensates for the potential decrease in the resistance to pitting caused by oil contamination and increases the resistance slightly.

In case of the uncoated tribosystems, the dust in the oil affects the roughness of the wear track. It can be seen that the dust in the oil, due to its abrasive action, makes the worn surface rough and produces numerous surface defects, i.e., Sa increased from 0.05 to 0.10 μm, and Sz increased from 0.70 to 4.08 μm. These defects act like stress raisers and accelerate initiation of surface fatigue cracks in this way [43]; therefore, the lower resistance to pitting is, in the case of the steel–steel tribosystem, lubricated with the contaminated oil.

In case of the coating–steel tribosystem, it can be observed from EDS maps that the low-friction layer of WC/C and the hard, wear resistant interlayer of WC were worn away (due to abrasive action of the contaminant in the oil), exposing the adhesive layer of CrN. Due to high pressure in the contact zone, the material of the cone, together with the ductile CrN layer, were plastically deformed, and the width and depth of the wear track much increased compared with the uncoated tribosystems. It reduced the high maximum Hertzian pressure, reducing also the subsurface tangential stress. Therefore, there were fewer tendencies to fatigue cracks initiated beneath the surface, and of pitting occurrence.

In summary, from the results obtained with the W-DLC/CrN-coated 18CrNiMo7-6 steel specimens lubricated with contaminated PAO oil, it can be stated that the presence of the coating gave the best resistance to the three modes of investigated wear, abrasion, scuffing, and pitting. Although, in previous tests, the coating reduced the resistance to pitting, a very important observation is that, in case of contaminated oil, pitting was mitigated rather than accelerated. This is very important when taking into account the very extreme working conditions of mining conveyors, in which oil contamination by coal or lignite dust occurs.

## 4. Summary and Conclusions

The research aimed at extending the lives of the gears in the global transmissions of chain and belt conveyors in the mining industry.

We presented the tribological behaviour of two antifriction coatings deposited on case-hardened 18CrNiMo7-6 steel. Model simple specimens were used in the tests.

Two types of thin, antifriction coatings were tested: W-DLC and W-DLC/CrN, representing an a-C:H:Me group. In the abrasion and scuffing tests, the vee blocks were coated, leaving the test pins uncoated. In the pitting tests, the test cones were coated. Three material combinations were tested in that way: (W-DLC)-steel, (W-DLC/CrN)–steel, and steel–steel for reference.

Two commercial industrial gear oils were selected for lubrication, a mineral and a synthetic one, the latter having a PAO base, both with a viscosity grade of VG 320.

In comparison with the uncoated specimens, both the tested coatings improved the resistance to abrasion and scuffing, and in the same way. However, the W-DLC/CrN coating exhibited better resistance to pitting than did W-DLC, although both coatings reduced the resistance to pitting in comparison with the uncoated tribosystem. Thus, for the second phase of the experiments, the W-DLC/CrN coating was selected as the most-suited for use with 18CrNiMo7-6 case-hardened steel.

The results obtained with the W-DLC/CrN-coated 18CrNiMo7-6 steel specimens lubricated with PAO oil contaminated with the dust obtained from a coal mine allow us to state that the presence of the coating gave the best resistance to the three modes of investigated wear, abrasion, scuffing, and pitting. Although, when using pure oils, the coating reduced the resistance to pitting, a very important observation is that, in case of the contaminated oil, pitting is mitigated rather than accelerated.

Following the results of the tests, for the case-hardened 18CrNiMo7-6 steel dedicated to manufacture gears in the global transmissions of mining conveyors, the optimum coating is W-DLC/CrN. It gives the best resistance to abrasion, scuffing, and pitting, even when lubricated with the contaminated oil. This is very important when taking into account the very extreme working conditions of mining conveyors responsible for oil contamination by coal or lignite dust.

The next stage of the experiment will be the verification (gear) testing of the selected material combination, with special attention paid to lubrication by a contaminated oil.

## Data Availability

Not applicable.
